# Continuous effects of language ability and relative proficiency on bilingual children’s production of four advanced syntactic constructions

**DOI:** 10.1017/S1366728926101321

**Published:** 2026-05-06

**Authors:** Javier Jasso, Anny Castilla-Earls, Amanda Owen Van Horne

**Affiliations:** 1 Department of Spanish and Portuguese, The Ohio State University, Columbus, OH, USA; 2 Department of Speech, Language, and Hearing, The University of Texas at Dallas, Richardson, TX, USA; 3 Communication Sciences and Disorders Department, University of Delaware, Newark, DE, USA

**Keywords:** complex syntax, syntactic development, atypical language, DLD, subject and object relative clauses, passives, conditional adverbials

## Abstract

Current approaches to bilingualism and language learning ability obscure differences between capacity for learning (ability) and dominance (relative proficiency). Bilingual children with developmental language disorder (DLD) have persistent difficulties with complex syntax. The effects of language learning ability and relative proficiency on syntactic development in bilingual acquisition are not well described. This cross-sectional study examined the continuous effects of language ability and relative proficiency on the production of conditionals, subject/object relatives and passives in a sample of 34 five- to nine-year-old Spanish–English bilingual children, 12 of whom were identified as having DLD. Conditionals were significantly easier than other forms, and there were no differences between subject and object relatives. Higher language ability was associated with greater accuracy. Relative proficiency predicted higher English performance for balanced and English-dominant children. Further examination of language ability and relative proficiency in diverse language learners is warranted.

## Highlights

- Continuous effects of ability and relative proficiency examined.
- Language ability predicted accuracy across structures.
- Relative proficiency predicted differences between English and Spanish.
- Conditional items were easier than subject/object relatives and passives.
- Passives were easier in English than in Spanish.

Individual variability is central to bilingualism research. The last decade has seen calls to move away from a monolingual standard and study the variability within bilingual speakers (e.g., Rothman et al., 2023). Indeed, more continuous (rather than categorical) approaches to bilingualism are needed to advance the field (Xia et al., 2025). In speech, language and hearing research, children with developmental language disorder (DLD: difficulties with learning, understanding and using language that are not explained by other conditions, such as autism or hearing loss, or lack of input in a language; Bishop et al., 2016) are conventionally compared with typically developing (TD) peers, although there, too, is a growing recognition that language ability exists on a continuum (Kapantzoglou et al., 2015). Dichotomizing variables that are conceptually continuous for simplicity leads to an information loss and less power to detect effects (Royston et al., 2006). Thus, modeling language ability and language proficiency as continuous predictors is not only more statistically sound, but it also has the potential to contribute to a better understanding of how gradual differences in experience and ability influence language knowledge and use.

These continuous effects can be examined in the realm of complex syntax, which begins early but continues to develop well into the school-age years (Diessel, 2004). Crucially, bilingualism does not appear to affect the timing of complex syntax: Bilingual children show comparable complex syntax to monolingual children (Korade et al., 2024). However, language development associated with general language learning ability (i.e., TD vs. DLD) and language proficiency (i.e., in a particular language) may influence children’s complex syntax. This study examines the contribution of these two predictors to the complex syntax produced by a sample of Spanish–English bilingual children that includes both TD children and children with DLD. It is known that acquiring two or more languages in early childhood is not associated with an elevated risk of DLD, nor does it exacerbate its effects (Marini et al., 2025). It does, however, change its presentation: Just as TD bilingual children are qualitatively different from their monolingual peers, so, too, does the manifestation of language disorder in bilingual children differ qualitatively from monolinguals with DLD (De Houwer & Ortega, 2018). Thus, our aim was to study how continuous differences in language ability and language proficiency are related to the production of advanced syntactic forms. By advanced syntax, we refer here to various constructions that involve 1) embedding (e.g., subject relative clause: *the fish_AGENT_ that is splashing the duck_PATIENT_*), 2) noncanonical patient–agent word order (e.g., passive: *the frog_PATIENT_ was kissed by the princess_AGENT_*), or both (e.g., object relative clause: *the penguin_PATIENT_ that the bear_AGENT_ is scrubbing*).

## 1. Language ability and relative proficiency in bilingual children

In bilingual populations, it is necessary to distinguish between individuals’ underlying language learning skills (i.e., language ability) and their proficiency in a particular language, and to measure both adequately (De Houwer & Ortega, 2018). Language ability refers to general language learning capacity, which can be conceptualized and measured continuously in monolingual and bilingual children. This continuum, spanning below-average to above-average skills, represents all children, including those with typical language skills (low-average to above-average language learning skills) and those with DLD (below-average). In contrast, Language proficiency refers to linguistic competence in a particular language and is greatly affected by children’s experiences with that language. Relative proficiency or dominance captures a bilingual speaker’s performance in both of their languages and ranges from strongly dominant in one of their languages (i.e., differentials that are positive toward Spanish; Spanish dominant), balanced performance in both, or strongly dominant in the other language (i.e., differentials that are positive toward English; English dominant). Most children in the U.S., including those in our study, may start out dominant in the home language and experience a shift in this pattern following school entry (Castilla-Earls et al., 2019). However, the degree of dominance is not categorical. There is also a range of ability at each level of relative proficiency. For example, there are “balanced” bilingual children (i.e., differential close to 0) at low and high language ability levels. For this reason, we focus on relative, rather than single-language, proficiency.

Ability and relative proficiency are rarely studied together and are often conflated (Peña et al., 2021). This is perhaps unsurprising, since they have been developed from different fields (e.g., communication disorders vs. second language acquisition) for different purposes (i.e., identifying children with disorders vs. categorizing bilingual speakers). While differences in language ability due to neurodevelopmental differences can be detected by preschool age and remain relatively stable across time (Reilly et al., 2010), proficiency and dominance are positively correlated with measures of language exposure/use in children (Bedore et al., 2012). Both can shift more rapidly than language ability in response to changes in linguistic and social demands, such as school entry or a summer immersion (Castilla-Earls et al., 2019; Peña et al., 2021).

Language proficiency and language ability can be difficult to differentiate. Tools developed to assess ability can mistakenly diagnose low ability when proficiency in the tested language is low, particularly if the tool was developed for use in monolingual children. To decouple language ability from language proficiency, multiple sources of information are required, including standardized norm-referenced measures in both languages. For language ability, existing bilingual measures with appropriate diagnostic accuracy may be used to compute a best-language score that considers performance in both English and Spanish (Anaya et al., 2016). Single-language proficiency has conventionally been measured using rating scales or with direct behavioral measures, including measures of receptive vocabulary. Receptive vocabulary closely relates to other measures of proficiency and has been used to capture proficiency in previous studies (Paradis et al., 2022). Additionally, it taps language experiences and is less sensitive to clinical status. This is evidenced by the fact that these measures are not good indicators of impairment, with children with DLD routinely scoring in the low-average range (Anaya et al., 2018). Relative proficiency is the resulting difference in scores between the two languages and can be measured by comparing single-language receptive vocabulary scores to create a differential (Peña et al., 2021). Thus, the use of best-language scores and a vocabulary differential has the advantage of differentiating language proficiency from language ability in bilingual children. That is, a measure that examines language ability in both languages can be used to determine overall language skills using a best-language approach, while vocabulary measures in both languages can be used to determine the differential between the languages to measure language dominance.

## 2. Complex syntax in children with DLD

Studies of bilingual children’s production accuracy regularly use relative proficiency as a grouping variable to account for differences across bilingual experiences (e.g., Rothman et al., 2016), but they do not typically consider differences in language learning ability. To consider effects of language ability, we review what is known about bilingual children with DLD^1^, a language-based disorder characterized by below-average language performance across multiple domains that is not the result of reduced language experience or other neurodevelopmental profiles (Bishop et al., 2016, 2017). A large body of research focused on morphological performance has shown that mono- and bilingual children with DLD have significant morphological difficulties (Castilla-Earls et al., 2023). At the same time, some TD bilinguals may show some of these same production patterns when tested a few years after acquiring English (Meir, 2018).

Children with DLD exhibit difficulties acquiring the syntactic properties in their ambient languages, which has long-lasting impacts on their social and academic achievement. For instance, complex syntax makes up approximately one-third of the sentences in first-grade science texts and teacher scripts (Curran, 2020), suggesting broad academic implications. Weaknesses in complex syntax have also been found in children with DLD who are English-speaking monolinguals (Frizelle & Fletcher, 2014; Montgomery et al., 2017), speakers of other languages (Delage et al., 2024; Georgiou & Theodorou, 2022) and bilinguals in various language pairs (Gutierrez-Clellen, 1998; Meir, 2018; Paradis et al., 2022). These children take longer to acquire, and are less accurate at producing, advanced syntactic structures (Georgiou & Theodorou, 2022). The available studies that have compared TD–DLD bilingual children suggest that complex syntax may be a relative strength in bilingual children with average language ability, even when tested in their weaker language (Meir, 2018; Paradis et al., 2022). It remains unclear which structures are most difficult for bilingual children with DLD. Knowledge of performance patterns in both typical and atypical populations may help identify older school-age children and inform treatment, which is currently lacking.

## 3. Construction accuracy in English and Spanish

In this study, we examine children’s complex syntax using elicited productions of four syntactic constructions in English and Spanish: subject/object relative clauses, passives and conditional adverbial clauses. These structures have similar form–meaning correspondences in both languages (e.g., similar constituent ordering and uses in discourse), which may facilitate production. Previous work has shown that even partly overlapping structures are accessed as if representationally similar (Kotzochampou & Chondrogianni, 2022). At the same time, some structures, such as passives, may have different input frequencies (Gámez & Shimpi, 2016; Green, 1975). We briefly review the characteristics of these structures and what is known about the developmental trajectories of these constructions in both monolingual and bilingual children across language ability, including children with and without DLD.

### 3.1. Conditional adverbial clauses

Complex sentences with a conditional adverbial clause and a main clause are pragmatically useful for communicating cause–effect relationships and hypothetical reasoning. Conditional clauses can be sentence-initial (*if you finish, you can play*) or sentence-final (*you can play if you finish*). Both languages exhibit a preference for initial if-clauses (Park, 2002), unlike most adverbials, which are ordered after the main clause. For example, this is attested in English language corpora, where the initial position is used approximately three-fourths of the time (Park, 2002). One explanation that has been posited is iconicity effects, which refers to a perceived resemblance between form and meaning. Iconicity effects suggest that ordering these elements as they occur temporally, since the conditional precedes the outcome, influences accuracy (De Ruiter et al., 2021). In naturalistic environments, children are often first exposed to conditionals that are prohibitive (*if you don’t X, you can’t Y*). While studies have found a comprehension–production asymmetry, a variety of adverbials – particularly causal, purposive and conditional adverbials – are found early in children’s language productions (De Ruiter et al., 2018; Diessel, 2004).

In children with DLD, spontaneous production studies of adverbial clauses (i.e., not limited to conditionals) have produced mixed findings, such that children with DLD produce these clauses at lower or similar rates to TD children. In her study of Spanish–English bilingual children, Gutierrez-Clellen found similar rates of adverbial production between TD children and a group of teacher-identified “low achievement” children (Gutierrez-Clellen, 1998). A more recent study of bilingual children acquiring English with various home languages showed that bilingual children with DLD used significantly fewer adverbial clauses (Paradis et al., 2022).

### 3.2. Relative clauses

Restrictive relative clauses are adjectival clauses that modify a noun phrase (Kroeger, 2005). Relatives in English and Spanish are head-initial (i.e., postnominal) and primarily use a gapping strategy (Kroeger, 2005). Relative clauses can be subclassified by the syntactic role of the gap: Subject relative clauses gap the subject of the clause (NP *that* _ V NP: *the seal that saw the dog*), while in object relative clauses, the object is gapped (NP *that* NP V _: *the seal that the dog saw*). These two constructions are similar across English and Spanish syntax (Castilla-Earls & Owen Van Horne, 2023). Still, there are potential differences in their linear order, the grammaticality of resumptive pronouns and the degree to which a relativizer is obligatory. Spanish has freer word order, such that postverbal subjects (VS) are possible and grammatical. Subject-topicalized object relative clauses (SV) match the English word order, while subject inverted relatives (VS) are the less marked option (Llompart & Dąbrowska, 2024). In addition to the gapping strategy, a resumptive pronoun strategy is available for object relative clauses (the seal_i_ that the dog saw *him_i_ / la foca_i_ que la_i_ vio el perro) across the Spanish-speaking world (De Mello, 1992). Finally, English object relatives can have a null relativizer, but this is ungrammatical in Spanish (the seal (that) the dog saw / la foca *(que) el perro vio).

Compared with subject relative clauses, object relative clauses have a greater distance between the head and the gap and exhibit noncanonical patient–agent word order. Previous work consistently shows that object relative clauses are later acquired (see Lau & Tanaka, 2021, for a review), more difficult to process for child and adult speakers (Kidd et al., 2007; Llompart & Dąbrowska, 2024), less frequent in spontaneous data (Diessel, 2004; Macdonald et al., 2020) and less accurate in elicited production (Ezeizabarrena, 2012). Several studies have found an advantage of subject relatives in spontaneous and elicited production, as well as higher accuracy in sentence repetition paradigms (Ezeizabarrena, 2012; Kidd et al., 2007; Wada et al., 2020). Children often begin with relative clauses that express a single proposition. For example, Diessel (2004) found that most English-speaking children’s relative clauses were presentational (e.g., *This is the sugar that goes in there*). As noted by Diessel (2004), the earliest relative clauses produced by children (subject relatives with intransitive verbs) differ considerably from those routinely tested. Object relatives often occur with a prototypical patient or theme (e.g., *the song he recommended; the book I read*). To minimize the possibility that users are relying on their knowledge of prototypical agents (e.g., animate, likely human) and patients (inanimate), studies have used semantically reversible relatives, where either NP can be assigned agent or patient roles. For example, in the relative clause *the fish that is splashing the duck*, either animal could be initiating the splashing. These reversible relatives substantially increase difficulty (Kidd et al., 2007; Macdonald et al., 2020). Monolingual children in Kidd et al. (2007) repeated more sentences containing subject relatives than those with object relatives when both contained animate NP heads, but this difference disappeared when object relatives contained inanimate heads.

Children with DLD have shown lower rates of relative clause production than TD peers. Attempted relative clauses in children with DLD may be characterized by omission of obligatory relativizers in subject relatives (Hesketh, 2006), production of reduced relatives (e.g., *the monkey hanging on the tree*: Hesketh, 2006) or avoidance strategies. These children also have more difficulty with object relatives than subject relatives, perhaps to a greater extent than that seen in TD children. Some studies have found that children with DLD have more difficulty with multiple propositions than a single proposition in a presentational context (Frizelle & Fletcher, 2014). While we are not aware of large-scale studies regarding the trajectories for bilingual children with DLD, similar difficulties can be expected.

### 3.3. Passives

Verbal passives are periphrastic structures available in both languages (NP *be* V-ed *by* NP) to communicate a patient-focused event. In Spanish, the verbal passive is not the most common passive construction^2^ (Gámez et al., 2009) and is late acquired in monolingual children (Gámez et al., 2009), compared with other complex syntax. However, there is evidence from priming studies that these two passive constructions are represented together in bilingual speakers (Kotzochampou & Chondrogianni, 2022). The passive is formed in both languages through noncanonical patient–agent word order and periphrastic constructions that use an auxiliary (*be* or *get* and *ser* or *estar*). In Spanish, this includes gender/number agreement between the subject and the past participle (Armstrong & Montrul, 2025). Production studies typically test long passives, which include by-phrases headed by a preposition (*by* or *por*) that introduce the agent. As with relatives, passives that are semantically reversible are often used to investigate structural accuracy.

Studies of bilinguals’ production of verbal passives point to various factors relating to accurate production (Armstrong & Montrul, 2025; Gámez & Shimpi, 2016; Rothman et al., 2016). The difficulty with passives is frequently attributed to difficulties in thematic role assignment posed by the noncanonical word order. As with relative clauses, animacy influences both processing ease and production accuracy. Bilingual studies have pointed to several possibilities that drive accuracy, including relative proficiency (i.e., low levels of language exposure), low construction-specific input (Gámez et al., 2009) and cognitive/maturational effects (Rothman et al., 2016). Gámez and Shimpi (2016) tested 6-year-old Spanish-dominant bilingual children on full Spanish passives. Results from their picture description task showed that children did not produce any long passives spontaneously (study 1). When the same task was adapted for a priming paradigm, a similar sample of children showed a priming effect when compared with those exposed to active primes (study 2). In a recent study of an older sample of children, Armstrong and Montrul (2025) found effects of Spanish literacy instruction on accurate repetition of Spanish sentences with verbal passives in English-dominant bilingual children: Children who attended bilingual programs produced more reversible verbal passives than those in English-only schools. In a different bilingual context of Spanish-dominant Colombian children attending an English-immersion school, students’ age, and not their English exposure, was significantly associated with greater longitudinal growth of English passive comprehension using a forced-choice paradigm (Rothman et al., 2016). Overall, these studies offer explanations related to input effects (increased exposure to literacy or input-specific exposure boosting performance) and maturational effects (with older children making more gains when exposure was held constant).

Children with DLD, like younger TD children, experience difficulties interpreting passives, relying instead on semantics or word order (Montgomery et al., 2017). Although much of the evidence available focuses on comprehension, there is reason to believe that similar difficulties cascade into production (Delage et al., 2024). Using elicited production tasks, several studies have documented difficulties in children with DLD speaking English, Cantonese and Mandarin (Durrleman et al., 2024).

Existing studies of children’s spontaneous and elicited production indicate that various linguistic factors condition production of conditionals, subject/object relative clauses and passives. Reasons previously identified for why these are difficult to produce include embedding (with a dependent clause) and canonicity (i.e., conforming to the most frequent word orders present in the language). Canonical structures tend to be easier in production for typical and disordered populations across the lifespan (Lau & Tanaka, 2021), and children with DLD have difficulty with structures that require embedding. These structures under discussion have similar structural properties, which allows us to examine effects of ability and relative proficiency considering both languages.

## 4. Summary and research questions

The summarized research suggests that difficulties associated with complex syntax production in bilingual children may be due to low language ability (as in the case of children with DLD), low proficiency in a particular language, or both. It remains unclear whether overall language learning ability or relative proficiency predicts learning of such advanced language forms in both languages. It is also unclear which specific structures are most affected by ability or relative proficiency levels. We asked the following research questions:

1. Does the effect of language ability on production accuracy vary by construction type?

We predicted effects of language ability and construction type, as well as a language ability x construction type interaction. Given the documented difficulties with complex syntax in children with low language ability, including bilingual children with DLD (Meir, 2018; Paradis et al., 2022), overall language ability (as indexed by the best-language score on a bilingual test of English/Spanish language disorder) was predicted to positively predict syntactic accuracy. We predicted an effect of construction type based on canonicity such that noncanonical frames (i.e., passives, object relative clauses) would be less accurate than canonical frames (i.e., conditionals, subject relative clauses). We also predicted an interaction between ability and construction type, such that children with lower language ability would have a more pronounced difference between noncanonical and canonical frames (Georgiou & Theodorou, 2022; Kidd et al., 2007).

1. Does the effect of relative proficiency on production accuracy vary by language, and does this effect vary by construction type?

We predicted within-language dominance effects revealed as an interaction between relative proficiency and language such that relative proficiency in a particular language (as indexed by differences between single-word receptive vocabulary standard scores across English and Spanish) would positively predict an advantage in accuracy of production in that language. That is, Spanish-dominant children were expected to have a Spanish advantage, with greater accuracy on Spanish items than English items. Likewise, English-dominant children were predicted to have an English advantage. Previous studies have found effects of relative proficiency or exposure on complex syntax in spontaneous speech and in comprehension (Paradis et al., 2017; Scheidnes & Redmond, 2023). While we expected this interaction to be affected by the specific construction type, we did not have a strong prediction regarding which constructions would show greater or smaller effects of relative proficiency on item language.

## 5. Method

All study procedures were overseen by the Institutional Review Board at University of Houston. Data from this study were collected just prior to the COVID-19 pandemic, and the research questions and analyses were preregistered in Open Science Framework: https://doi.org/10.17605/OSF.IO/SYJGK.

### 5.1. Participants

Data for this study include responses from 34 five- to nine-year-old Spanish–English bilingual children who had a mean age of 79.24 months (*SD* = 12.23; range: 59–111). Children in this study are a subset of children participating in a longitudinal study examining bilingual development. Children were recruited from the community and public schools in the greater Houston area. To be eligible for the longitudinal study, children had to understand both Spanish and English, per parent report. In addition, children passed an otoacoustic emission hearing screening, indicating normal hearing, and obtained a score of ≥ 70 on the Matrices subtest of the *Kaufman Brief Intelligence Test – Second Edition* (KBIT-2; Kaufman, 2004), indicating nonverbal IQ skills within normal limits. There were no group differences on the KBIT-2, matrices subtest between TD children (*M* = 102.32, *SD* = 11.58, range = 83–123) and those with DLD (*M* = 111.08, *SD* = 13.95, range = 92–145), *t*(19.38) = −1.86, *p* = .08. All children included in the longitudinal study were eligible to participate in the present study. However, because of COVID-19 restrictions, only those children who were tested in person (before March 2020) were included. Of 44 eligible children tested, five children with data in only one language were excluded (Spanish: *n* = 3; English: *n* = 2). An additional five children were removed because they had limited exposure (i.e., only during school) to one language: Four children were third-/fourth-/fifth-generation with limited exposure to Spanish, and the remaining child was born outside of the U.S. and had limited exposure to English. This restricted the sample to 34 children.

DLD status was determined at time 1, and all other data presented here were collected during time 2. At time 1, 12 of these children were considered to have DLD. Identification of DLD status was done through a converging evidence approach, which is currently the gold-standard procedure for identifying DLD in bilingual children. Under this approach, TD and DLD status is determined through triangulation of various sources of data, including parent/teacher report, spontaneous language and standardized scores (see Castilla-Earls et al., 2020). Use of this approach enhanced our confidence of accurate classification. At time 2, there was still a wide range of language ability in this sample, as measured by the BESA or BESA-ME best-language score (mean standard score of 94.59; *SD* = 15.83; range: 60–120).

Demographic information came from caregiver responses at time 2. Descriptive statistics of the entire sample can be found in Table 1. In terms of relative proficiency, children had a mean vocabulary differential of 3.50 (*SD* = 17.46, range = −30–55), indicating that, at the level of the sample, there was slight Spanish dominance. All children were reported to be Hispanic, and most children were reported to be White (*n* = 26). Other responses to race included no response (*n* = 3) and “other” (*n* = 5). Over half (*n* = 23, 68%) of children were second-generation immigrants (i.e., first in their family to be born in the U.S.). The remaining children were reported to be third generation (i.e., parents were born in the U.S.: *n* = 8; 24%), fifth generation^3^ (i.e., parents, grandparents and great-grandparents were born in U.S.: *n* = 1; 3%), or did not respond (n = 2; 6%). Parents’ country of origin included Mexico (*n* = 16), mainland U.S. (*n* = 13); Puerto Rico (*n* = 1) and “other” (*n* = 4). Mother’s highest level of education was reported to be elementary school (*n* = 8), high school (*n* = 7), some college (*n* = 3), associate’s degree (*n* = 3), bachelor’s degree (*n* = 6) and graduate degree (*n* = 7). Most children (*n* = 30, 88%) attended dual-language or bilingual programs in the greater Houston area; the rest attended English-only (*n* = 3) or Spanish immersion programs (*n* = 1).

**Table 1.** Participant demographic information and language performance in means (standard deviations) as measured at time 2

Measure	M (SD)
*N*	34
Sex	19 female
Age, months	79.24 (12.23)
PPVT	86.00 (17.43)
TVIP	89.50 (19.87)
Relative proficiency	3.50 (17.46)
KBIT–2	105.41 (12.97)
English BESA/BESA-ME	89.88 (18.99)
Spanish BESA/BESA-ME	81.04 (19.09)
Language ability	94.59 (15.83)

*Note*: For all language measures, standard scores are reported. For relative proficiency, positive numbers indicate more Spanish. Language ability was calculated based on *each child’s best*-language BESA or BESA-ME standard scores. KBIT-2 performance was based on year 1. BESA, Bilingual English–Spanish Assessment (Peña et al., 2018); BESA-ME, Bilingual English–Spanish Assessment—Middle Extension (Peña et al., 2008); KBIT-2, Kaufman Brief Intelligence Test*–*Second Edition; PPVT, Peabody Picture Vocabulary Test, Fourth Edition; TVIP, Test de Vocabulario en Imágenes Peabody.

Parents provided consent at the beginning of the longitudinal study, and children provided assent at the beginning of each testing session, which was carried out at school or at home. Language ability, Spanish and English proficiency and all experimental tasks were completed by two Spanish–English bilingual examiners over two separate sessions, with one completing all testing in English and the other in Spanish. During the Spanish testing session, children were spoken to exclusively in Spanish and were encouraged to respond in Spanish. The same approach was used during the English sessions. The language tested first was randomized at the participant level. General language/cognitive assessments (discussed below) were completed first, and then probes were completed and included both the syntax probes reported here and other morphological probes.

### 5.2. Measures

#### 5.2.1. Language ability

Language ability was assessed using the Bilingual English–Spanish Assessment (BESA; Peña et al., 2018) for children ages 4;0 to 6;11 or the Bilingual English–Spanish Assessment – Middle Extension (BESA-ME; Peña et al., 2008) for those aged 7;0 to 10;11. Best-language scores, regardless of the language of the score, were used to index language ability. This approach is useful for distinguishing cases in which a bilingual child has stronger language skills in one language from cases where the child has low language skills in both languages, which qualifies them as having DLD. Rather than classifying children as TD/DLD, standard scores in the best language were employed as a predictor variable. Although two language ability groups are clinically useful, there is empirical evidence for a range of language abilities (Kapantzoglou et al., 2015), and a more continuous approach allows for better estimates of the effects.

#### 5.2.2. Single-language and relative proficiency

Single-language proficiency was measured using the English and Spanish versions of the Peabody (Peabody Picture Vocabulary Test, Fourth Edition [PPVT-IV], Dunn & Dunn, 2007; Test de Vocabulario en Imágenes Peabody [TVIP], Dunn et al., 1986). Relative proficiency was the difference score between standard scores in each language (Peña et al., 2021). This resulting continuous measure ranged from negative to positive values, with more positive values representing higher Spanish dominance, more negative values representing higher English dominance and values close to zero reflecting balanced proficiency.

### 5.3. Syntactic elicited production task

A researcher-developed elicited production task was used to test bilingual children’s English and Spanish knowledge of four syntactic forms. Specifically, children were administered 40 syntactic probe items that tested four parallel constructions in both languages (five items for four constructions in each language): conditionals, subject relative clauses, object relative clauses, and passives (Table 2). The items were the same in both languages, and each task included two training items that paralleled the experimental trials. All responses were audio recorded for offline transcription.

**Table 2.** Example of test sentences for each syntactic construction in Spanish and English

Syntactic construction	Description	Elicitation	Example
Conditional^a^	If-marked dependent clause with event & result main clause Spa: si [NP V (NP)] [NP V (NP)] Eng: if [NP V (NP)] [NP V (NP)]	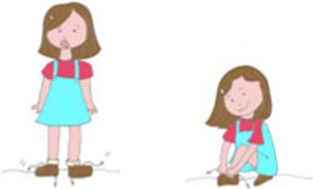 The mom tells the girl…	Si se te sueltan los cordones, amárrate los zapatos. If your shoelaces get loose, tie your shoes.
Subject Relative^a^	A subject-gapped relative clause modifying an animate NP head Spa: NP*_i_* [que Agent-_*_i_* V (a) Patient-NP*_j_*] Eng: NP*_i_* [that Agent-_*_i_* V Patient-NP*_j_*]	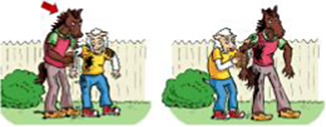 Who is the arrow pointing to?	Al caballo*_i_* que __*_i_* empuja al borrego*_j_.* The horse*_i_* that __*_i_* pushes/’s pushing the sheep*_j_.*
Object Relative^b^	An object-gapped relative clause modifying an animate NP head Spa: NP*_i_* [que Agent-NP*_j_* V Patient_*_i_*] Eng: NP*_i_* [(that) Agent-NP*_j_* V Patient-_*_i_*]	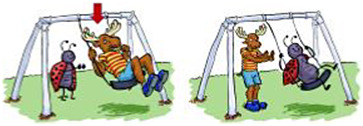 Who is the arrow pointing to?	Al alce que empuja __*_i_* la mariquita. The moose (that) the ladybug pushes/is pushing __*_i_.*
Passive^b^	A semantically reversible passive construction with a by-clause. Spa: Patient-NP*_i_* auxser V-ada/o _*_i_* [por Agent-NP*_j_*] Eng: Patient-NP*_i_* auxbe V-ed _*_i_* [by Agent-NP*_j_*]	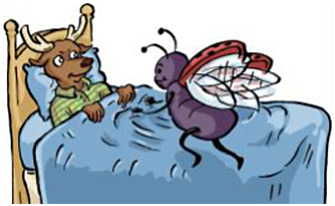 What is the bug doing to the deer? Start with the deer. The deeri…	Fue despertado __*_i_* por el bicho*_j_.* Was woken up __*_i_* by the bug_j_.

*Note*: Examples shown are practice items. Eng, English; NP, noun phrase; Spa, Spanish; V, verb.

a Canonical word order.

b Noncanonical word order.

Conditional adverbials were elicited in an imperative sentence by asking children to provide advice to a child in the picture. Children were shown a panel of two pictures that depicted the conditional element on the left (e.g., raining outside) and the outcome on the right (e.g., a person taking an umbrella). They were told to provide advice to the children pictured from the mother’s perspective using the pictures (The mom says to the girl/boy ___.). For instance, Table 2 shows a girl with her shoes untied, and the same girl tying her shoes. The conjunction *if* or *si* was written between the two pictures, and children were trained to describe both panels using a sentence that included this word. Responses were prompted to describe the pictures (What’s going on here?), elicit a pragmatically appropriate imperative (But what does the mom tell the boy?) and include the target (Start with *if.*). Conditional utterances were marked as correct if they included one dependent if-clause, one independent clause, evidence of embedding and correct order of events. The conditional clause could be sentence-initial or sentence-final. Sentences such as *If it’s raining, take your umbrella* received a score of 1 because they included all elements. Incorrect sentences included when-clauses (*When it’s raining get an umbrella.*), single propositions (*Brush your hair if you brush your hair*) and unclear cause/effect relations (*Si te peinastes el pelo, la necesitas hacer* [If you brushed your hair, you need to do it]).

Subject and object relative clauses were tested in a presentational context with a single proposition to avoid additional cognitive demands (Diessel, 2004; Kidd et al., 2007). Embedded verbs for all items were transitive, actional and concrete (e.g., *splash*). Creating the appropriate felicitous context was important to support production of the appropriate type of relative clause. Both subject and object relatives were elicited by showing two near-identical picture scenes of animals acting upon each other, where the only difference was the agent and patient. For example, children saw two pictures: one of a horse pushing a sheep and the other a sheep pushing a horse (Table 2). An arrow was used to bring focus to the relevant NP head (agent or patient), and the child was asked, “Which animal is the arrow pointing to?” For subject relative clauses, the arrow pointed to the horse agent to elicit the target structure (*the horse that’s pushing the sheep*). For object relative clauses, the arrow pointed to the sheep patient (*the sheep the horse is pushing*).

To be considered correct, subject relative clauses required a lexical NP head + overt relativizer + embedded clause with both an agent and a patient (Kroeger, 2005). Subject relatives with resumptive pronouns or NPs in either language (e.g., *the cat who it chased the dog*) were considered incorrect, as these attempts provided insufficient evidence of a relative clause. Similarly, object relative clauses required a head noun, a relativizer and an embedded clause. In English, object relatives did not require an overt relativizer (e.g., *the dog that/∅ the cat is chasing;* Kroeger, 2005), while in Spanish the overt relativizer *que* was obligatory. Given word order in Spanish, object relatives such as *the dog the cat chased* could be produced with a preverbal subject (SV order), as in English (e.g., *el perro que el gato persigue ___*), or with a postverbal subject (VS order) (e.g., *el perro que persigue ___ el gato*). Oblique and genitive relative clauses were accepted in Spanish (e.g., *a un poodle que un ratón le está cepillando el pelo [a poodle_i_ who a mouse is brushing him_i_ the hair]*). Passive relative clauses (e.g., *the dog that was chased by the cat*) were also accepted as correct if they were semantically appropriate, even though they were not the intended target. Finally, resumptive pronoun strategies were accepted for English object pronouns (e.g., *the toad_i_ that the seal kissed him_i_*) and Spanish direct or indirect object clitics (*una hormiga_i_ que un zorro lo_i_ está abrazando [an ant_i_ that a fox is hugging him/it_i_]*). Children were given up to two prompts for each trial. If the child did not respond, they would be given a prompt that included both animals (There’s a __ and a __. What’s going on?). If the child only gave the NP with no relative clause (e.g., *the dog*, *that one*), they were prompted to specify with a pragmatic reason (But which one? There’s two.).

All passive items contained reversible animate NPs (i.e., animals). A patient-oriented question was used to prompt the child to describe the action starting with the patient. For example, the child would hear the examiner say, “What is the bug doing to the deer? Start with the deer.” (Target: *the deer was woken up by the bug*). Children were given up to two prompts. Responses were prompted if they contained an active construction (Start with the deer, OK?) or with one-word responses (Can you tell me the whole thing?). Passive constructions were correct if they included the correct subject-as-patient mapping, periphrastic construction and by-phrase with the agent; thus, short passives were not given credit. The following additional considerations were taken: In addition to *be*-passives, children could produce *get*-passives, which are more common in spoken registers (Montag & MacDonald, 2015). In Spanish, *ser* or *estar* passives were accepted, provided they were long passives. Variation in periphrastic passives and preposition selection that was attributable to cross-linguistic influence (English to Spanish or Spanish to English) was accepted, including *agarrar* passives (i.e., with the lexical verb *agarrar*, presumably by analogy of get-passives: *El ratón está agarrando limpiado por el pollito* [The mouse is getting cleaned by the chick]), *from/for*-phrases in English (*The frog is getting kissed from a rat*) and *de*-phrases in Spanish (*La abeja está siendo empujada de un venado [A bee is being pushed de-preposition a deer]*). Nontarget structures such as active sentences, se-passives, function-passives with se- and an external argument (*El ratón se limpia por el pollo [The mouse se washes by the chicken]*), and sentences with a fronted object (e.g., *El sapo lo está besando la foca [The toad, the frog_i_ is_i_ kissing him]*) were not given credit.

### 5.4. Transcription and scoring

All responses were transcribed verbatim by bilingual research assistants and then scored by the second author, a native Spanish speaker. Transcribers and scorers did not have knowledge of children’s clinical status, ability scores or proficiency. Scoring focused on the production of the target syntactic frame; therefore, responses with minor nontarget grammatical errors (e.g., article errors) and nontarget vocabulary (e.g., lexical substitutions for the animals) were accepted (e.g., Armstrong & Montrul, 2025). Scoring reliability was ensured using a double-scoring approach. The first author, without knowledge of DLD status, age or language scores, reviewed the scoring of all items by construction type and language. Cases of disagreement or discussion were highlighted and discussed between the first and second authors in a single face-to-face session. Overall, 1,340 items were reviewed, 71 items were flagged for discussion, and 35 of these resulted in a changed score. Primary disagreements included *estar* passives in Spanish (*n* = 11), unclear two-propositional conditionals (*n* = 5) and stative relative clauses (*n* = 5).

Code-switched utterances in which the nontarget language was used for an entire utterance or clause (e.g., *if it’s raining, lleva tu paraguas*) were not given any points to ensure that we could separate production in both languages. In contrast, those utterances with intrasentential code switching where either the NP or the VP was switched but not both (e.g., *if the hand is dirty, wash their manos*) were scored for accuracy. Partially intelligible utterances (i.e., one or more unintelligible segments) were included and scored for accuracy if they contained a clear syntactic frame that could be determined based on the intelligible segments (e.g., *if the boy was so hot, drink some X; if X make a mess, go sweep the floor*). Fully unintelligible utterances and no responses following multiple prompts were scored as incorrect.

## 6. Analytic plan

All statistical analyses and visualizations were conducted in R version 4.2.2 and RStudio 2022.12.0 (R Core Team, 2022). Visualization used *ggplot2* package version 3.5.1. Preregistered analyses can be found here: https://doi.org/10.17605/OSF.IO/SYJGK. In addition, the raw data and R code can be found here: https://doi.org/10.17605/OSF.IO/6ANCB. To answer our research questions, we ran mixed-effects logistic regression models using the “glmer()” function in the *lme4* package version 1.1-31 (Bates et al., 2015). This generalized linear mixed model (GLMM) approach was appropriate given the binomial distribution of the outcome variable. Post hoc pairwise comparisons were tested using the “emtrends()” and “pairs()” functions from the *emmeans* package version 1.11.1 (Lenth, 2023), controlling for multiple comparisons. While our preregistered analysis plan did not include these post hoc comparisons, these estimates were added to allow us to answer our research questions more directly.

Models constructed for research questions 1 and 2 were similar. Item accuracy (0/1) was the outcome variable. Language ability (best-language BESA/BESA-ME standard score) and relative proficiency (difference score between TVIP and PPVT standard scores) were entered as continuous variables to allow for better estimates of these effects and scaled using *z*-scores. Random effects for all models included child- and item-level intercepts; random slopes were not included due to power considerations. Categorical variables in our models (construction type, item language) used effects coding (or deviation coding), which uses the grand mean as the intercept in the model and avoids difficulty with interpretation that comes with other coding approaches. Models were allowed to run longer than the default setting using the “glmerControl()” function and the BOBYQA optimizer. This allows the model 10 times more time than the default setting to find the optimum and prevents premature stopping. All relevant contrasts to our research questions were tested using post hoc comparisons for the specified models.

To answer the first research question regarding the effects of language ability and construction type, we tested fixed effects for language ability, age, construction and two two-way interactions: language ability x construction and age x construction. Age was included to account for developmental effects. Language of the item was not considered since this was not of interest for this research question. The standardized estimates of age and ability were evaluated to compare their relative effects within the model. The following R formula was used: accuracy ~ construction * (ability + age) + (1|participant) + (1|item). To test for effects of construction type, we calculated the estimated marginal means for all pairwise comparisons (i.e., six in total) and then tested these effects using the “pairs()” function. To test for ability- and age-related effects and their interactions, we examined estimated marginal means representing slopes across different levels of construction and tested whether any of these were significantly different from one another.

The second research question asked whether children’s relative proficiency predicted differences in accuracy between English and Spanish performance and whether construction type influenced this effect. Fixed effects included relative proficiency, construction, language of the item (English or Spanish) and the three-way interaction of relative proficiency x item language x construction. We modified the preregistered formula in order to include this three-way interaction, with the following formula: accuracy ~ construction * item language * relative proficiency + (1|participant) + (1|item). While it converged, the three-way interaction was not significant; furthermore, a likelihood-ratio test showed that it was not significantly different from a model without this interaction, 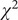 (2) = 2.08, *p* = .557. Thus, this interaction term was removed, and we proceeded with the more parsimonious model that tested all possible two-way interactions: relative proficiency x construction, relative proficiency x item language, and construction x item language. Given this dropped interaction term, we focused on the relative proficiency x language construction, leaving aside the question of differential effects by construction. Once this interaction was dropped, the following formula was used: accuracy ~ (construction + item language) * relative proficiency + construction * item language + (1|participant) + (1|item).

The continuous variable of relative proficiency, based on the differences in receptive vocabulary scores, had more positive values (*M* = 3.50; *SD* = 17.46; median: 1.00), indicating a slightly Spanish-dominant sample. While there were no outlier scores, one child had a much higher relative proficiency score (differential score of +55; +2.95 SD above the sample mean). Of particular concern, given the extremity of this score, was whether this child is representative of the population and whether their results are generalizable. An examination of this child’s scores indicated plausible values across demographic variables aligned with this dominance profile, indicating that they are representative of this population and their results are likely generalizable. Nevertheless, two models with identical parameterization were conducted, one with the full dataset (*n* = 34) and one that excluded this Spanish-dominant child (*n* = 33).

## 7. Results

### 7.1. Descriptive performance

We ran Pearson correlations to examine linear associations between age, language ability and relative proficiency. All three correlations were nonsignificant: Age was weakly associated with language ability, *r*(32) = .24, *p* = .179, and with relative proficiency, *r*(32) = −.12, *p* = .508. Language ability and relative proficiency were not associated with one another, *r*(32) = .04, *p* = .816, suggesting that these are indeed mapping onto different constructs.

In total, children produced 68 code-switched responses (4.84% of total), 116 scorable partially intelligible items (8.25%), 63 fully unintelligible utterances (4.48%) scored as incorrect, 147 non-responses (10.46%) and 8 “don’t know” responses (0.57%). Table 3 provides a summary of children’s performance (percent accuracy) on the four constructions (conditional, subject relative, object relative and passive) in English and Spanish. Overall, children were slightly more accurate in English than in Spanish. Conditional accuracy (55%) appeared to be more accurate than the other three constructions in both languages. Likewise, subject (Spanish: 16%; English: 18%) and object relative clauses (Spanish: 16%; English: 20%) showed remarkably similar performance in both languages. Passives patterned differently across languages: In English, passives were 28% accurate, the second most accurate construction. In contrast, in Spanish, passives were 8% accurate and ranked the least accurate construction.

**Table 3.** Syntactic accuracy (proportion correct), in means and standard deviations, by construction type and item language

Syntactic construction	Spanish	English	All
Conditional	0.56 (0.38)	0.54 (0.42)	0.55 (0.39)
Subject relatives	0.16 (0.27)	0.18 (0.34)	0.17 (0.31)
Object relatives	0.16 (0.31)	0.20 (0.36)	0.18 (0.33)
Passives	0.08 (0.15)	0.28 (0.39)	0.18 (0.31)
All constructions	0.24 (0.34)	0.30 (0.40)	0.27 (0.37)

*Note*: 20 items missing for Spanish data.

### 7.2. Effect of language ability

Our first research question asked whether children with higher language ability were more likely to produce accurate productions of complex syntax after accounting for developmental effects. The following terms were significant: construction, ability and age x construction. Constructions differed in their overall accuracy; post hoc comparisons showed that, overall, conditionals were significantly more accurate than subject relatives (*z* = 7.77), object relatives (*z* = 7.50) or passives (*z* = 7.77; all *p*s < .0001), which did not differ significantly from each other (all *p*s > .05).

**Figure 1.**
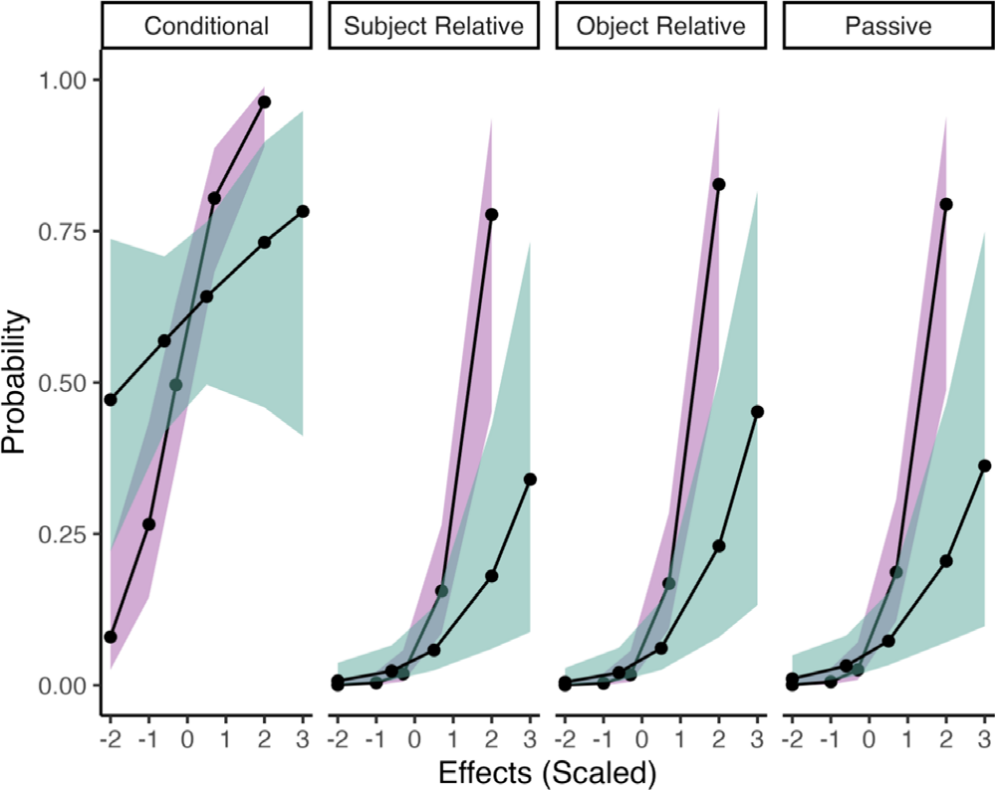
Predicted effects of language ability (purple) and age (green) on construction.

There was a significant and positive effect of language ability across constructions (Table 4). While an ability x construction interaction was detected in the main model, this effect was no longer significant when controlling for multiple comparisons, 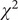 (3) = 6.19, *p* = .103. Age significantly predicted accuracy across constructions, and the effect of age was significantly larger for object relatives than for conditionals (*z* = –2.64, *p* = .042), which showed a flatter slope (Figure 1). Finally, there was a larger effect of ability in this model, OR = 7.97, 95% CI [4.30–14.75], compared with the effect of age, OR = 2.08, 95% CI [1.33–3.26]. Predicted accuracy across age and ability by construction is shown in Figure 1. Construction contrasts and age and ability slopes are shown in Supplementary Tables S1, S2 and S3.

**Table 4.** Logistic regression model results for the effects of language ability, construction type and age

Coefficient	Estimate	SE	CI	Statistic	*p*
(Intercept)	0.10	0.03	0.06–0.18	−7.70	**<0.001**
Construction1	14.74	3.80	8.90–24.43	10.45	**<.001**
Construction2	0.37	0.13	0.19–0.72	−2.91	**.004**
Construction3	0.36	0.13	0.18–0.73	−2.88	**.004**
zAbility	7.97	2.51	4.30–14.75	6.60	**<.001**
zAge	2.08	0.47	1.33–3.26	3.22	**.001**
Construction1 × zAbility	0.52	0.14	0.31–0.87	−2.48	**.013**
Construction2 × zAbility	1.21	0.41	0.62–2.33	.56	.575
Construction3 × zAbility	1.43	0.50	0.72–2.85	1.03	.303
Construction1 × zAge	0.63	0.11	0.46–0.88	−2.72	**.007**
Construction2 × zAge	1.12	0.19	0.81–1.55	.71	.478
Construction3 × zAge	1.32	0.23	0.95–1.85	1.65	.099
**Random effects**					
*σ* ^2^	3.29				
*τ*_00 id_	1.25				
*τ*_00 typenumber_	0.07				
*N*_id_	34				
*N*_typenumber_	20				
Observations	1307				
Marginal *R* ^2^/conditional *R* ^2^	0.638/0.741				

*Note:* *P*-values are based on Wald *z*-tests from the generalized linear mixed-effects model. Bold values indicate *p* < .05.

### 7.3. Effects of relative proficiency on English and Spanish performance

Our second research question sought to test a within-subject language dominance effect by exploring the role of relative proficiency (i.e., language dominance) on the differences between accuracy in English and Spanish. There was a nonsignificant three-way interaction among relative proficiency × item language × construction, 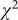 (3) = 2.18, *p* = .536 (Supplementary Figures S1 and S2). The following terms were significant: construction, item language, construction × item language and relative proficiency × item language (Table 5). Post hoc tests were used to understand the specific effects.

**Table 5.** Logistic regression model results for the effects of relative proficiency, item language and construction type for full dataset (*n* = 34)

Coefficient	Estimate	SE	CI	Statistic	*p*
(Intercept)	0.14	0.06	0.06–0.32	−4.73	**<.001**
Construction1	12.00	2.63	7.81–18.43	11.35	**<.001**
Construction2	0.41	0.08	0.27–0.61	−4.36	**<.001**
Construction3	0.50	0.10	0.34–0.74	−3.50	**<.001**
Language1	1.32	0.12	1.10–1.58	3.00	**.003**
zRelProf	1.23	0.50	0.55–2.71	.50	.615
Construction1 × language1	0.63	0.09	0.47–0.83	−3.31	**.001**
Construction2 × language1	0.80	0.13	0.59–1.09	−1.41	.159
Construction3 × language1	0.87	0.13	0.64–1.17	−.93	.350
Construction1 × zRelProf	0.81	0.13	0.59–1.10	−1.38	.169
Construction2 × zRelProf	1.31	0.23	0.93–1.85	1.53	.126
Construction3 × zRelProf	1.07	0.19	0.76–1.52	0.40	.686
Language1 × zRelProf	0.79	0.08	0.66–0.96	−2.41	**.016**
**Random effects**					
*σ* ^2^	3.29				
*τ*_00 id_	4.93				
*τ*_00 typenumber_	0.10				
*N*_id_	34				
*N*_typenumber_	20				
Observations	1307				
Marginal *R* ^2^/conditional *R* ^2^	0.231/0.696				

*Note:* *P*-values are based on Wald *z*-tests from the generalized linear mixed-effects model. Bold values indicate *p* < .05.

**Figure 2.**
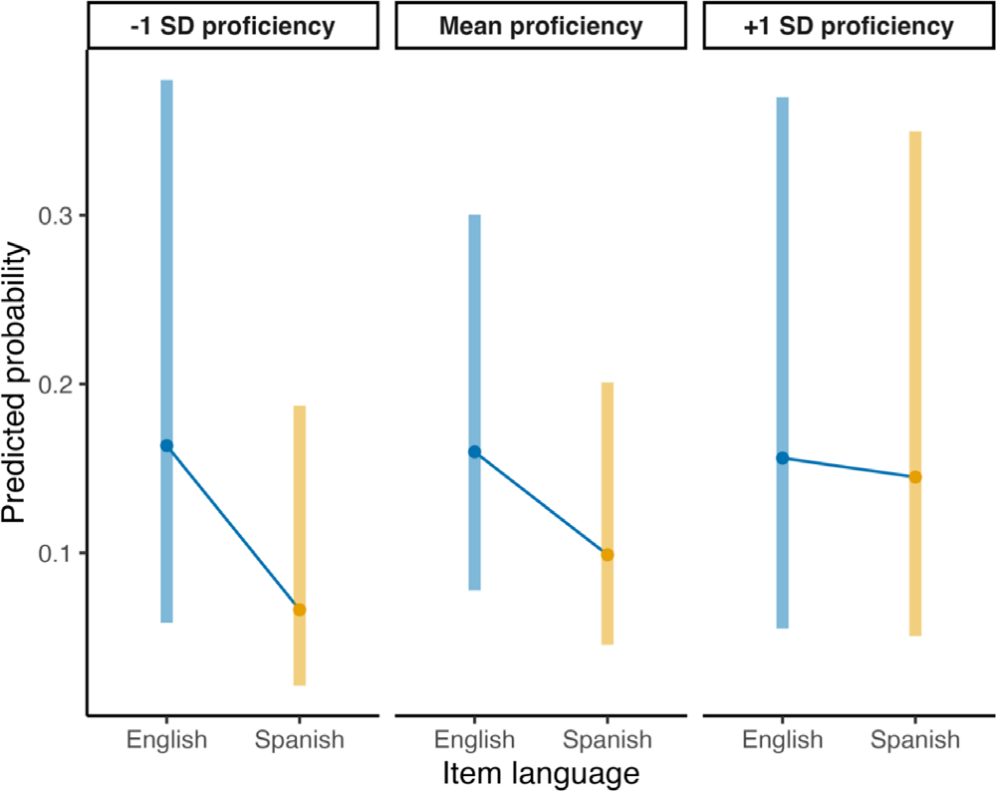
Predicted differences between English and Spanish at varying levels of relative proficiency scores (*z*-scores) for full dataset (*n* = 34).

The significant interaction between relative proficiency and item language can be interpreted to mean that while relative proficiency did not influence English and Spanish individually, children’s relative proficiency score influenced the degree to which their Spanish and English performance differed. While the effect of individual slopes was not significant, the difference in slopes between English and Spanish was statistically different, indicating the Spanish slope is more positive than the English slope, *z* = 2.46, *p* = .014. We examined this interaction effect further by statistically testing the English–Spanish slope difference at three levels of the continuous relative proficiency variable to represent English-dominant, balanced and Spanish-dominant profiles (i.e., at –1, 0 and +1 SD from the mean, respectively). This is shown in Supplementary Table S4. English and Spanish slopes were significantly different at –1 SD (*z* = 3.74, *p* < .001) and the mean (*z* = 3.00, *p* = .003) but not at +1SD, *z* = 0.34, *p* = .735. Predicted differences in accuracy by relative proficiency profiles are shown in Figure 2.

A construction × item language interaction was driven by differences between English and Spanish passives, such that the English passives were more accurate, *z* = 5.37, *p* < .0001. No other difference was detected between languages.

**Figure 3.**
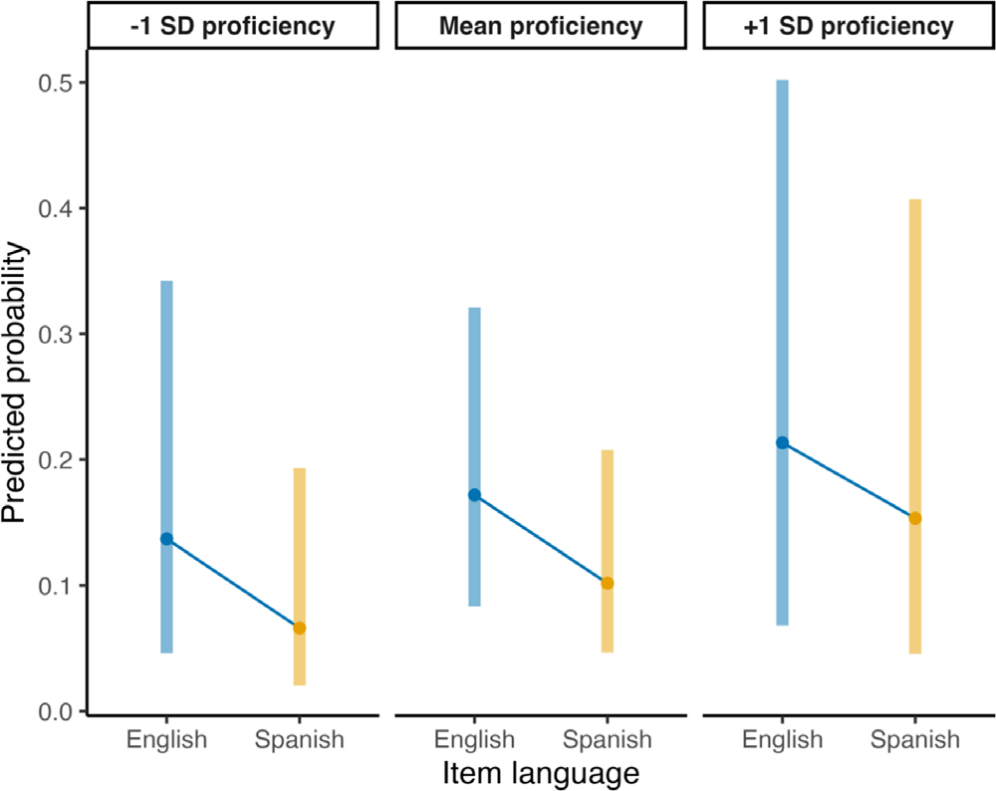
Predicted differences between English and Spanish at varying levels of relative proficiency scores (*z*-scores), excluding Spanish-dominant child (*n* = 33).

Recall that one participant had a relative proficiency profile that was much more strongly Spanish dominant than other children (i.e., vocabulary differential of +55). Accordingly, we carried out analyses both with and without this participant (Table 6 and Figure 3). The pattern of results was largely similar; however, the model with the participant excluded (*n*=33) showed a nonsignificant interaction of relative proficiency x language, *z* = −.90, *p* = .367. As before, the three-way interaction was nonsignificant, 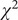 (3) = 4.26, *p* = .234.

**Table 6.** Logistic regression model results for the effects of relative proficiency, item language and construction type, excluding Spanish-dominant child (*n* = 33)

Coefficient	Estimate	SE	CI	Statistic	*p*
(Intercept)	0.15	0.06	0.07–0.35	−4.53	**<.001**
Construction1	11.27	2.50	7.30–17.40	10.93	**<.001**
Construction2	0.42	0.09	0.28–0.64	−4.15	**<.001**
Construction3	0.50	0.10	0.33–0.74	−3.46	**.001**
Language1	1.35	0.12	1.13–1.62	3.29	**.001**
zRelProf	1.45	0.68	0.57–3.65	.78	.435
Construction1 × language1	0.67	0.10	0.51–0.89	−2.75	**.006**
Construction2 × language1	0.76	0.12	0.56–1.04	−1.71	.088
Construction3 × language1	0.86	0.13	0.64–1.17	−.93	.350
Construction1 × zRelProf	0.69	0.14	0.47–1.02	−1.85	.065
Construction2 × zRelProf	1.59	0.34	1.05–2.41	2.17	**.030**
Construction3 × zRelProf	1.02	0.21	0.69–1.52	.11	.909
Language1 × zRelProf	0.90	0.10	0.73–1.13	−.90	.367
**Random effects**					
*σ* ^2^	3.29				
*τ*00 id	4.85				
*τ*00 typenumber	0.10				
*N*id	33				
*N*typenumber	20				
Observations	1268				
Marginal *R* ^2^/conditional *R* ^2^	0.233/0.694				

*Note:* *P*-values are based on Wald *z*-tests from the generalized linear mixed-effects model. Bold values indicate *p* < .05.

## 8. Discussion

Our study used a continuous approach to bilingualism (i.e., relative proficiency) and language ability to understand their effects on children’s elicited productions of conditionals, subject relatives, object relatives and passives. This study is unique in the continuous approach employed here, testing various constructions across both languages in the same sample, allowing us to detect age-, ability- and dominance-driven changes in production. We saw effects of language ability across all four construction types. Age also affected the accuracy of subject relatives, object relatives and passives, albeit to a lesser degree than language ability. Conditionals had significantly higher accuracy than the other constructions and had a smaller effect of age compared with object relatives. Relative proficiency predicted children’s better language across constructions, but further investigation is needed. Passives elicited in English were significantly more accurate than those elicited in Spanish.

### 8.1. Construction accuracy

Construction type drove children’s accuracy. Specifically, we observed a clear advantage of conditionals over the other three constructions. In both languages, it was easier for children to produce a conditional adverbial plus a main clause than other forms. As a reminder, conditional items were command forms in a specific pragmatic situation (i.e., giving advice). Irrealis or hypothetical conditionals, which are marked with subjunctive in Spanish, are likely to be more challenging, but were not elicited here. De Ruiter et al. (2018) show that hypotheticals, event plausibility, and iconicity affect production in English, suggesting that our results may not extend to less temporally salient or less concrete propositions. Additionally, children were given the word to use to promote the use of *if* rather than other conjunctions (e.g., *when, while and then*), which may have further promoted accuracy. That said, this finding may also be due to an advantage of producing adjunct clauses external to the main VP, which are more similar to conjoined clauses (Diessel, 2004). Regardless of the reason for this effect, it remains unclear if this observed conditional advantage is part of a broader adverbial advantage over both complements and other adjuncts such as relative clauses. To verify this possibility, additional adverbial forms would need to be tested since conditionals are unique in other ways. For instance, they occur preferentially in the preposed or initial position, unlike most other adverbials, which are more likely to occur in a postposed position (Diessel, 2004).

Particularly relevant to our initial questions, this study does not provide support for the idea that canonical structures are easier to produce. Although conditionals were highly accurate, subject relative clauses were not more accurate than other forms and, in particular, were not significantly different from object relatives in either language. There may also be dissociable differences in their learnability: In a treatment study conducted by Castilla-Earls et al. (2023), children produced low rates of conditional and subject relatives in both languages at pretest, but only conditionals saw gains following the intervention. Canonicity advantages reported in previous studies may be related to the fact that canonical sentences tend to be simpler overall or may only appear in comprehension (Montgomery et al., 2017). Studies on language acquisition in English suggest that task factors such as animacy and plausibility may be critical to accuracy (e.g., De Ruiter et al., 2018; Macdonald et al., 2020). Exploration of corpus data suggests that a variety of semantic and event-related cues affect difficulty of production. We only tested a single type of each structure. Future work should explore whether accuracy varies with item manipulations.

Low accuracy (i.e., less than 20%) of both subject and object relatives in both languages was an unexpected finding. Previous production studies, which have focused on younger monolingual children, show that relative clauses and relative clause constructions (i.e., main clause and embedded relative clause) are present in spontaneous production and elicited production (Diessel, 2004; Ezeizabarrena, 2012; Kidd et al., 2007). As a reminder, all relative clauses elicited were semantically reversible using various animals as NPs. Accurate productions required the NP head signaled by the picture, a relativizer (except for English object relatives), an embedded verb and NP with an appropriate thematic role (agent or patient). We did not penalize for lexical substitutions (e.g., *poodle* for *sheep*); however, this may have still caused production difficulties for low-frequency animals (e.g., *moose*). Because multi-propositional relatives are harder than single propositions (e.g., presentational context), we focused only on the relative clause, which likely lowered the cognitive demands. Still, the relative clauses expected here required two full NPs that were semantically reversible. This is in contrast to other common relatives seen in production, such as intransitive subject relatives (*the dog that’s barking*) and object relatives with a pronoun (*the sandwich I like*). Indeed, children followed these patterns, producing relative clauses with intransitive stative verbs, ambiguous relative clauses (*al pollito que está atacando [the little chicken who is attacking/who it’s attacking]*), topicalization and passivized relatives (Ferreiro et al., 1976, as cited in Torrens, 2024). Studies of younger children in English and various Romance languages have also seen a preference for passivized relatives (Guasti & Cardinaletti, 2003). The overall low accuracy makes it difficult to detect effects attributable to variation in age, ability or relative proficiency. These low rates in production across both languages also do not allow us to examine potential differences in production patterns. For example, omission of relativizers in subject relatives, a pattern reported in U.S. English-speaking children, may be more common in English than in Spanish in the same children. Longitudinal data collection would allow detection of age and relative proficiency effects as accuracy increases (e.g., Rothman et al., 2016, for passives).

The passive advantage observed for English items is likely due to differences in input frequency. The same children exposed to the same items produced passives in English (28%) but not in Spanish (8%). Spanish allows for various passive structures, with the one tested here being most often used in written or formal registers. In fact, it may be that this type of clause is not regularly produced by young bilingual speakers who have not yet had extensive exposure to written text (Gámez & Shimpi, 2016). Spanish corpus studies show limited or virtually absent use of passives in children’s spontaneous speech (Gámez et al., 2009), and very low frequency of the long passive across Spanish registers (Green, 1975). Higher passive rates are expected with exposure to written text (Montag & MacDonald, 2015). In Armstrong and Montrul (2025), a sample of slightly older English-dominant bilingual children (8–12 years) produced Spanish verbal passives of the type *Anoche fue llevado el chico por la mamá* (Last night the boy was carried by the mom) with 36-40% (English-only) to 81-86% accuracy (bilingual instruction); differences in the task (sentence repetition vs. elicited production in our study) and semantic plausibility may explain these divergent findings. In our task, children were told to start with the semantic patient for each picture, which almost certainly reduced simple transitive sentences as a viable option. Instead, children used various strategies, often with nontarget complex syntax (e.g., complement clauses or adverbials), to maintain the subject in question and the embedded clause to be in the canonical order. For example, one child used an object relative clause (*The pig está mirando a la rana que la está observando [The pig is looking at the frog that’s watching it]*). Children’s responses also contained the target copula verbs and by-phrases to express meanings that are more frequently expressed in both languages, such as progressive aspect, locative phrases, and prepositional phrases (Armstrong & Montrul, 2025). Thus, when verbal passives are not readily available to these language users, constructions with shared formal aspects may be recruited.

### 8.2. Language ability

Children’s language ability, as indexed by their best-language scores on the BESA/BESA-ME, was predictive of item accuracy, even after accounting for the developmental effects of age. This is in line with and extends previous work on complex syntax in DLD (Georgiou & Theodorou, 2022; Meir, 2018; Paradis et al., 2022). Critically, the scoring allowed nontarget errors in unrelated areas (e.g., verb morphology, agreement), which further points to deficits in complex syntax that are not the result of other grammatical difficulties. This ability effect holds in general across all forms tested, adding to the body of work showing ability-driven effects on complex syntax production. Our prediction that noncanonical constructions would be especially difficult for children with lower language ability was not borne out in the results, as there were no detectable differences in the influence of ability on accuracy (as shown by differences in slope) for each construction. Overall, this finding offers an important theoretical contribution to the DLD profile in different language learning contexts and suggests that further exploration of the contributions of different constructions, language combinations and learners is needed to understand the way that language ability and age interact with canonicity effects.

### 8.3. Relative proficiency on differences in accuracy between languages

This study also explored whether there was evidence of dominance effects for the same speakers. Previous research has shown that relative proficiency can affect language production in both of the speaker’s languages (Bedore et al., 2012; Dunn & Tree, 2009). Our results showed an effect of relative proficiency on differences in children’s complex syntax between languages. Even though our sensitivity analysis with and without the Spanish-dominant child indicated this interaction disappears when the child is excluded, we interpret the model with the significant interaction, albeit with caution. The results pointed to an English advantage when comparing children’s scores across both languages. Different levels of relative proficiency affected these differences in accuracy asymmetrically: English-dominant and balanced bilingual children had more accurate English performance than Spanish performance. This was not true of the Spanish-dominant children, who showed no such difference by language, as seen in Figure 2. Our sample was restricted by the COVID-19 pandemic, and we anticipate that if we had a more complete sample, including other children who were strongly Spanish dominant, we would observe this pattern of relative accuracy shifting with dominance more robustly. Additional work is required to verify these findings. These results suggest that productive syntax is sensitive to language dominance, similar to what has been reported in morphology or lexical semantics (Bedore et al., 2012; Dunn & Tree, 2009).

Across the board, English scores were higher than Spanish scores, but this was only significant for English-dominant and balanced children. We wondered why this sample would perform better in English, considering many of them (88%) received bilingual programming in the school setting. Two non-mutually exclusive explanations are linguistic factors (i.e., differences in difficulty of complex syntax constructions across languages) and children’s language experiences (i.e., their total and structure-specific input). While our design does not allow us to distinguish between these two possibilities, differences across English and Spanish in the passive construction point to linguistic factors. In terms of children’s bilingual experiences, this may also be related to the sociocultural dominance of English in Southeast Texas, where it is the language of government and media. This links to previous work showing greater-than-expected language performance in English-dominant language contexts (Brozgold & Centeno, 2007) and would also be consistent with the findings shown in Figure 3 that English accuracy is stronger for all dominance profiles.

In addition to dominance effects, we also considered the possibility of a three-way interaction with construction type. Such a finding would have indicated that these dominance effects differentially affected the four constructions tested. This effect was not detected in our models, although trends in different directions were observed (Supplementary Figures S1 and S2). Differences across structures may be more apparent when children have accuracy rates that are neither at floor nor at ceiling. Given the overall difficulty that children had with these forms, we cannot rule out floor effects masking effects of language dominance. Thus, further studies with a larger sample, a longitudinal sample and/or older children would be needed to make more precise claims about these relationships.

One limitation of this work is that data collection was interrupted by COVID-19, leading to a substantially smaller-than-planned sample. Thus, small effects are interpreted here but should be considered preliminary and worthy of additional study. Another implication of the small sample size is that we were unable to consider both relative proficiency and child language ability in the same statistical models; this is a question worthy of future examination in large samples. We also acknowledge other relevant predictors, such as nonverbal intelligence and SES, that were not entered in our models; the extent to which these may be predictive of complex syntax merits careful study in future work. Additionally, findings should be interpreted in the context of the community, with most bilingual children tested having relatively strong support for both languages in school/home contexts. These limitations notwithstanding, we believe these findings are informative given the limited data available about the production of complex syntax by Spanish–English bilingual children and the continuous effects of both relative proficiency and ability.

## 9. Conclusion

This study examined the production of complex syntax in a group of Spanish–English bilinguals, offering insight into acquisition patterns in children with varying levels of ability (including children with DLD) and relative proficiency during this developmental period. The children examined here showed significant ability-driven differences for conditionals, subject/object relative clauses and verbal passives. Conditional items, which have canonical word order and are more frequent in the input, presented less difficulty for all children and were less affected by age than object relatives. Passive items appeared to be especially difficult in Spanish. No difference between subject and object relative clauses emerged in either language, likely due to low accuracy rates. There was preliminary evidence of dominance effects on complex syntax performance for English-dominant and balanced children. Future work should examine these dominance effects using spontaneous language tasks. While elicited production tasks offer compelling evidence that children have tacit knowledge of these productions, spontaneous measures can complement these findings by revealing clause types in linguistic contexts that more closely align with input in both languages, as well as early attempts at complex syntax (e.g., Diessel, 2004). Efforts to examine spontaneous production of complex syntax in the same sample of children are currently underway. Further investigation into the comprehension and production of complex syntax structures in bilingual children with and without DLD would shed light on how cognition and experience shape children’s language use.

## Supporting information

10.1017/S1366728926101321.sm001 Jasso et al. supplementary material Jasso et al. supplementary material

## Data Availability

The data that support the findings of this study are available in Open Science Framework at https://doi.org/10.17605/OSF.IO/6ANCB.

## Footnotes

1 We draw on the literature using DLD and specific language impairment (SLI) to describe the patterns seen in children with language disorder but exclusively use the term DLD throughout, consistent with the CATALISE consortium recommendations (Bishop et al., 2016, 2017).

2 We elicited verbal passives in Spanish, which are parallel to the English construction and allow an external argument (i.e., by-phrase). The more frequent impersonal passive constructions marked by a *se* clitic do not allow an external argument.

3 The child reported as fifth generation was confirmed to be bilingual, with regular use of both languages and better language scores in Spanish, so they were included.

## Supplementary material

The supplementary material for this article can be found at http://doi.org/10.1017/S1366728926101321.
