# Supplementary material for: Continuous effects of language ability and relative proficiency on bilingual children’s production of four advanced syntactic constructions
Source: Biling (Camb Engl). 2026 May 6:1–16. Online ahead of print. doi: 10.1017/S1366728926101321 (PMC13221095; doi:10.1017/S1366728926101321)

Table S1. Pairwise Comparisons of Constructions

Contrast	*Odds Ratio*	*SE*	*df*	*null*	*z*	*p*
Conditional / Subject Relative	39.464	18.668	Inf	1	7.770	**0.000**
Conditional / Object Relative	40.562	20.036	Inf	1	7.496	**0.000**
Conditional / Passive	29.767	12.995	Inf	1	7.773	**0.000**
Subject Relative / Object Relative	1.028	0.591	Inf	1	0.048	1.000
Subject Relative / Passive	0.754	0.405	Inf	1	-0.525	0.953
Object Relative / Passive	0.734	0.405	Inf	1	-0.560	0.944

Note. *P*-values are Tukey-adjusted for multiple comparisons. Bold values indicate significant contrasts ($p < .05$).

Table S2. Pairwise Comparisons of Ability Slopes by Construction

Contrast	*Estimate*	*SE*	*df*	*z*	*p*
Conditional – Subject Relative	-0.834	0.478	Inf	-1.745	0.301
Conditional – Object Relative	-1.007	0.500	Inf	-2.013	0.183
Conditional – Passive	-0.742	0.443	Inf	-1.675	0.337
Subject Relative – Object Relative	-0.173	0.578	Inf	-0.299	0.991
Subject Relative – Passive	0.092	0.541	Inf	0.170	0.998
Object Relative – Passive	0.265	0.556	Inf	0.475	0.965

Note. *P*-values are Tukey-adjusted for multiple comparisons.

Table S3. Pairwise Comparisons of Age Slopes by Construction

Contrast	*Estimate*	*SE*	*df*	*z*	*p*
Conditional – Subject Relative	-0.572	0.272	Inf	-2.103	0.152
Conditional – Object Relative	-0.736	0.279	Inf	-2.636	**0.042**
Conditional – Passive	-0.511	0.264	Inf	-1.936	0.213
Subject Relative – Object Relative	-0.164	0.274	Inf	-0.599	0.932
Subject Relative – Passive	0.060	0.263	Inf	0.230	0.996
Object Relative – Passive	0.225	0.270	Inf	0.833	0.839

Note. *P*-values are Tukey-adjusted for multiple comparisons. Bold values indicate significant contrasts ($p < .05$).

Table S4. Pairwise Comparisons of Language Performance at Different Levels of Relative Proficiency

Contrast	*zRelProf*	*Odds Ratio*	*SE*	*df*	*null*	*z*	*p*
English / Spanish	-1	2.752	0.745	Inf	1	3.739	**0.000**
English / Spanish	0	1.733	0.318	Inf	1	2.995	**0.003**
English / Spanish	1	1.092	0.284	Inf	1	0.338	0.735

Note. *P*-values are Tukey-adjusted for multiple comparisons. Bold values indicate significant contrasts ($p < .05$).

Figure S1. Predicted Accuracy by Relative Proficiency and Construction

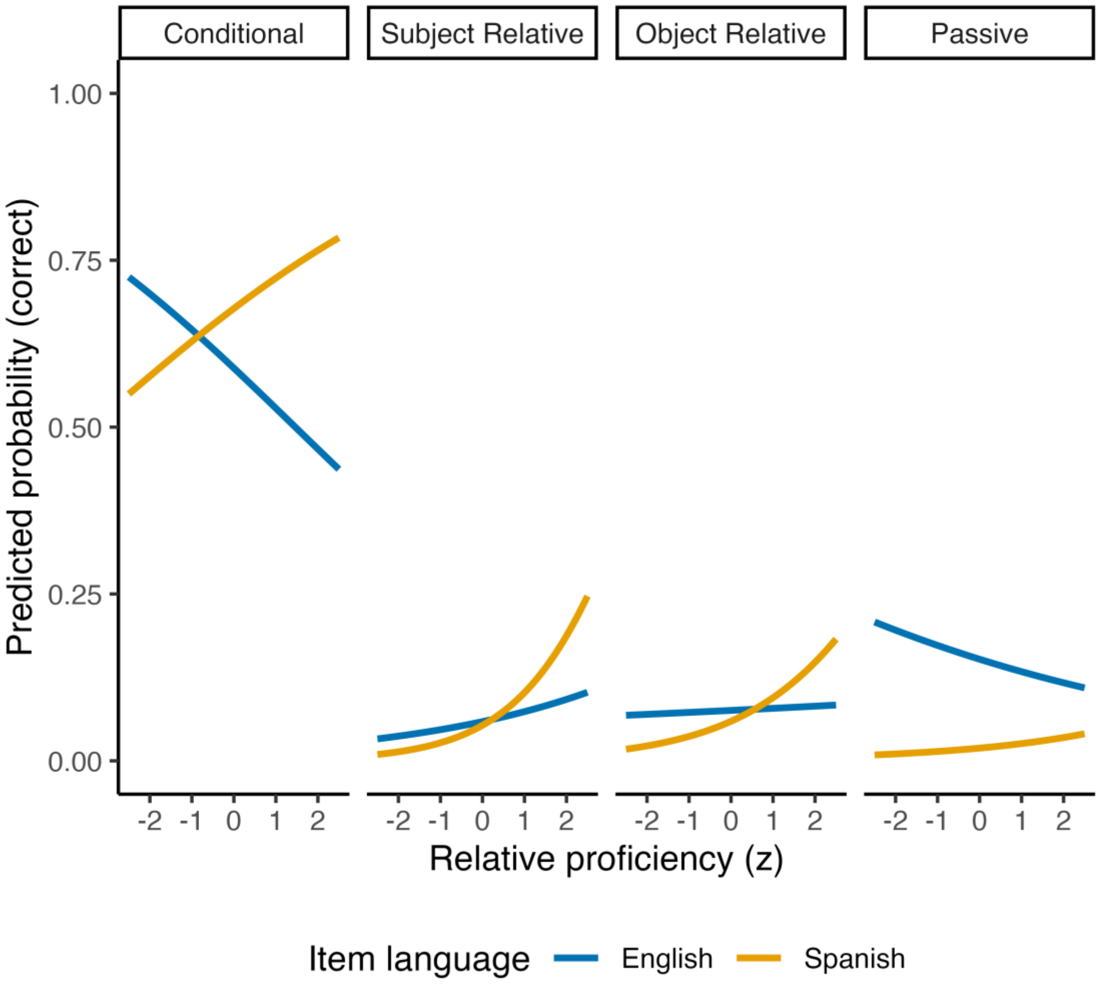

Figure S2. Predicted Accuracy by Relative Proficiency
and Construction (Without Spanish-Dominant Child)

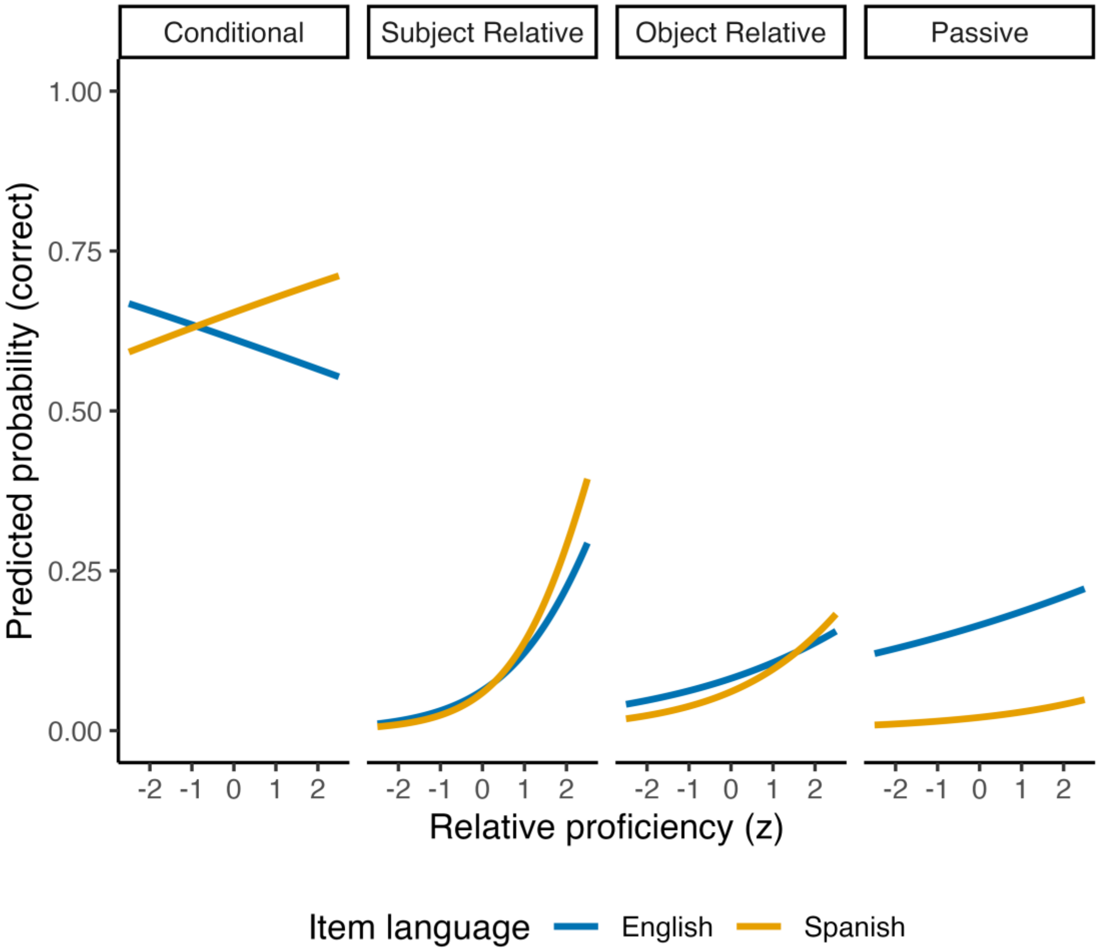

Supplement: Jasso et al. supplementary material [file S1366728926101321sup001.pdf]
